# Plastin 3 in health and disease: a matter of balance

**DOI:** 10.1007/s00018-021-03843-5

**Published:** 2021-05-23

**Authors:** Lisa Wolff, Eike A. Strathmann, Ilka Müller, Daniela Mählich, Charlotte Veltman, Anja Niehoff, Brunhilde Wirth

**Affiliations:** 1 Institute of Human Genetics, University of Cologne, Kerpener Str. 34, 50931 Cologne, Germany; 2 Center for Molecular Medicine Cologne, University of Cologne, Robert-Koch-Str. 21, 50931 Cologne, Germany; 3 Institute of Biomechanics and Orthopaedics, German Sport University Cologne, Cologne, Germany; 4 Cologne Center for Musculoskeletal Biomechanics, Medical Faculty, University of Cologne, Cologne, Germany; 5 Center for Rare Diseases Cologne, University Hospital of Cologne, 50937 Cologne, Germany

**Keywords:** Cutaneous T-cell lymphomas, Colorectal cancer, Osteoclasts, Amyotrophic lateral sclerosis, Ataxia

## Abstract

For a long time, PLS3 (plastin 3, also known as T-plastin or fimbrin) has been considered a rather inconspicuous protein, involved in F-actin-binding and -bundling. However, in recent years, a plethora of discoveries have turned PLS3 into a highly interesting protein involved in many cellular processes, signaling pathways, and diseases. *PLS3* is localized on the X-chromosome, but shows sex-specific, inter-individual and tissue-specific expression variability pointing towards skewed X-inactivation. *PLS3* is expressed in all solid tissues but usually not in hematopoietic cells. When escaping X-inactivation, PLS3 triggers a plethora of different types of cancers. Elevated PLS3 levels are considered a prognostic biomarker for cancer and refractory response to therapies. When it is knocked out or mutated in humans and mice, it causes osteoporosis with bone fractures; it is the only protein involved in actin dynamics responsible for osteoporosis. Instead, when PLS3 is upregulated, it acts as a highly protective SMN-independent modifier in spinal muscular atrophy (SMA). Here, it seems to counteract reduced F-actin levels by restoring impaired endocytosis and disturbed calcium homeostasis caused by reduced SMN levels. In contrast, an upregulation of PLS3 on wild-type level might cause osteoarthritis. This emphasizes that the amount of PLS3 in our cells must be precisely balanced; both too much and too little can be detrimental. Actin-dynamics, regulated by PLS3 among others, are crucial in a lot of cellular processes including endocytosis, cell migration, axonal growth, neurotransmission, translation, and others. Also, PLS3 levels influence the infection with different bacteria, mycosis, and other pathogens.

## Introduction

**Fig. 1:**
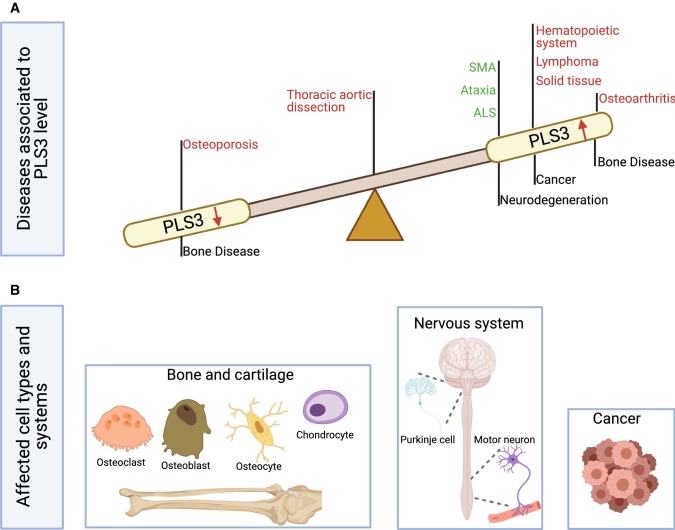
PLS3-associated disorders and main cell types involved. **a** PLS3 is involved in a variety of diseases, which associate with the PLS3 levels in a cell. Low protein abundance leads to osteoporosis, while increased levels are associated with cancer and osteoarthritis. In some neurodegenerative diseases, where F-actin levels are reduced, *PLS3* overexpression acts protective. Green letters imply a protective role of PLS3 while red letters highlight PLS3 as disease driving protein in the depicted disorders. *PLS3* plastin 3, *SMA* spinal muscular atrophy, *ALS* amyotrophic lateral sclerosis. **b** PLS3 fulfills distinct functions within different cell types. Osteoclasts, osteoblasts, osteocytes and chondrocytes are the target cells within the bone disease spectrum, which are influenced when PLS3 is dysregulated. In some neurodegenerative disorders, where motor neurons (e.g., SMA) or Purkinje cells (e.g., ataxia) are affected, overexpression of PLS3 showed a protective effect. Involvement of *PLS3* in cancer is highly divers and includes different kinds of solid tissues as well as hematopoietic and lymphatic cancers. The figure was created with BioRender.com

**Fig. 2:**
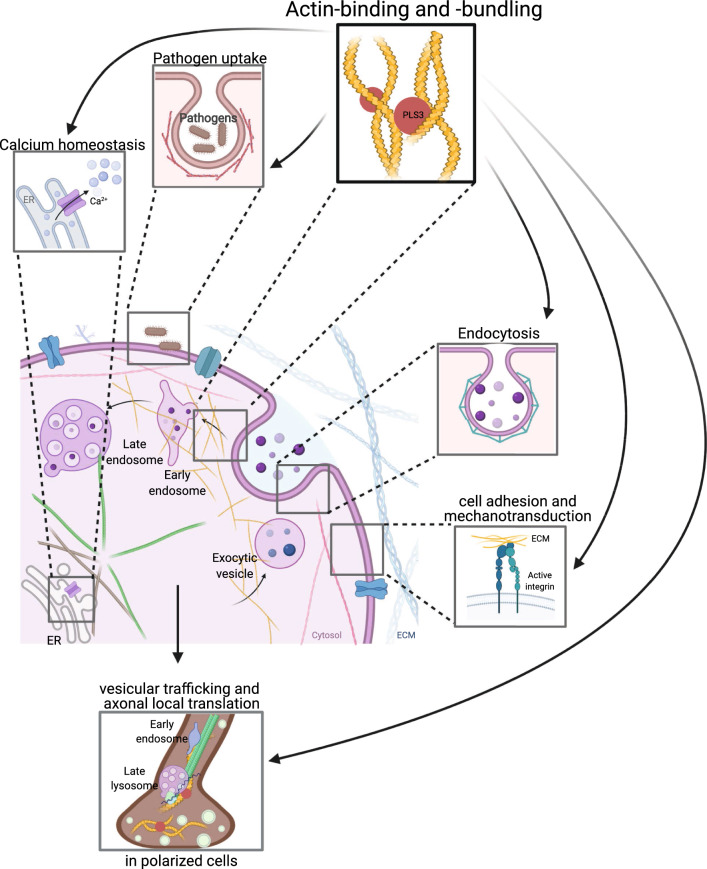
Cellular function of PLS3. The main function of PLS3 is its role in F-actin-binding and -bundling activity and, thus, in F-actin dynamics. Consequently, PLS3 is involved in endocytosis, cell motility, cell adhesion, mechanotransduction, pathogen infection, Ca^2+^ homeostasis, exocytosis, vesicle trafficking, axonal local translation and others. PLS3 protein is depicted as red circle. *ER* endoplasmic reticulum, *ECM* extra cellular matrix. The figure was created with BioRender.com

The ever-growing number of diseases, in which PLS3 is involved, highlights the importance of this F-actin-binding and -bundling protein, with a broad spectrum of cellular pathways (Fig. 1). It seems that the expression of *PLS3* is tightly regulated since knockout or mutations cause osteoporosis, while overexpression seems to trigger osteoarthritis and various types of cancer. Instead in several neuromuscular disorders, such as spinal muscular atrophy (SMA), amyotrophic lateral sclerosis (ALS) and *CHP1*-associated ataxia, PLS3 overexpression acts as a protective modifier. In this review, we present the current knowledge on the gene expression and protein function, the various cellular functions, in which PLS3 is involved and the disorders associated with PLS3 levels (decreased or increased) (Fig. 2).

## Gene and expression

In 1980, Bretscher & Weber identified fimbrin (later renamed to plastin), a 68 kD protein isolated from small intestine of the chicken [1]. Plastins are a family of actin-binding and -bundling proteins consisting of three tissue-specific paralogs *PLS1*, *PLS2*, and *PLS3* localized in humans on chromosomes 3, 13 and X, respectively [2]. The three isoforms share approximately 70% nucleotide sequence identity [3]. *PLS1* (plastin 1, I-plastin) is expressed in the intestine, colon, kidney and hair cell stereocilia of the inner ear, *PLS2* (plastin 2, L-plastin) in hematopoietic cell lineages and many types of cancer cells, and *PLS3* (plastin 3, T-plastin), the most abundant isoform, in cells from solid tissues [2, 3]. In lower organisms, such as yeast, only one isoform is present, known as fimbrin or Sac6. Before 2008, papers referred to fimbrin or T-plastin, and only after that, the correct name of plastin 3 has been established in publications.

The expression of *PLS3* is of utmost interest in the context of several medical conditions, such as spinal muscular atrophy (SMA), osteoporosis, osteoarthritis as well as a large number of cancers. While the regulation of the *PLS3* expression in different tissues is still elusive, several mechanisms have been proposed, which do not necessarily exclude each other (Table 1). Early reports stated that *PLS3* expression is limited to solid tissues [4]. However, elevated expression levels were found in blood samples in about 5% of healthy individuals [5]. *PLS3* shows high expression in the fetal and adult spinal cord and PC12 cells during neuronal differentiation, where it accumulates in growth cones [5]. *PLS3* mRNA is enriched and locally transcribed in the axonal compartment of motor neurons [6].

**Table 1:** Mechanisms that were proposed to regulate the expression of *PLS3* in cancer

Mechanism	Medical condition	Cell type	Effect	References
SNPs (*PLS3*) rs871773 C > T	Colorectal cancer	Circulating tumor cell	Low recurrence time	[84]
SNPs (*PLS3*) rs6643869	Colorectal cancer	Circulating tumor cell	Low recurrence time Sex-specific in women	[82] [83]
Copy number gain chromosomal instability	Colorectal cancer	Circulating tumor cell	Metastasis Poor prognosis	[79]
Expression associated with EMT	Breast cancer	Circulating tumor cell	Poor prognosis Especially triple-negative and Luminal A subtypes	[85] [86]
PLS3 triggers PI3K/AKT signaling	Pancreatic cancer	PACA cells	Poor survival	[75]
Non-small-cell lung cancer	Lung cancer		Poor survival	[91]
UV-resistance	Bladder, prostatic, head and neck cancer	Bladder, prostatic, head and neck cancer cells		[92, 93]
*PLS3* as downstream target of Lamin A	Colorectal cancer			[81]
Suppression of *PLS3* by *ZNF471*	Gastric cancer	Gastric cancer cells		[90]
Overexpression of *PLS3* by *LOXL1*	Gastric cancer	Gastric cancer cells		[88]
Promoter-specific hypomethylation	Sézary syndrome	CD4 +	Favorable disease outcome	[98]

*PLS3* is located in both, humans and mice, on the X-chromosome next to the macrosatellite *DXZ4* [7]. In women, approximately 15% of genes escape the X-chromosomal inactivation (XCI) and are, therefore, biallelically expressed [8]. Another 15% of genes variably escape XCI in a tissue-specific manner [8–10]. *PLS3* is a facultative escape gene in humans but not in mice [11]. The macrosatellite *DXZ4* is essential for XCI and has a highly variable repeat number of 50–100 copies of a 3 kb repeat monomer [12]. Interestingly, on the inactive X-chromosome, *DXZ4* is hypomethylated and binds to the transcriptional regulator protein CCTF-binding factor (CTCF), both features of chromatin regulation [13]. It has been hypothesized that the copy number of *DXZ4* may modulate the escape of genes in its molecular neighborhood and thereby the expression level of *PLS3* in females [14].

Accordingly, in *SMN1*-deleted asymptomatic women of spinal muscular atrophy (SMA) discordant families, the *PLS3* expression is elevated in blood and Epstein–Barr Virus transformed lymphoblastoid cells but not in their SMA-affected siblings, indicating a sex-specific protective effect (see more details in Sect. 3.6.1) [5]. Moreover, mutations or knockout of *PLS3* mainly affect men or male mice, while women or female mice are either very mildly or not affected, further supporting the sex specificity of *PLS3* expression [15, 16].

## Function

The main function of PLS3 is in F-actin-binding and -bundling. Consequently, PLS3 is involved in all processes dependent on F-actin dynamics such as cell motility, focal adhesion, cell division, endocytosis, neurotransmission, vesicle trafficking, axonal local translation, intracellular calcium PLS3-dependent processes, pathogen infection and others (Fig. 2).

### Protein structure

The three plastin isoforms seem to have different effects on the actin cytoskeleton organization depending on the cell type [17]. Plastins are monomeric proteins with a single polypeptide chain composed of two tandem repeats of actin-binding domains (ABD1 and ABD2) [18]. Each of these ABDs encompasses a pair of ~ 125 residue calponin homology (CH) domains (ABD1: CH1 and CH2, ABD2: CH3 and CH4) [19]. Each of the CH domains consists of four major α-helices. While three α-helices form a loose bundle of helices, the fourth is orientated perpendicular to the major bundles [20]. At their N-terminal end, plastins possess a Ca^2+^-binding regulatory domain (RD) of approximately 100 amino acids. The RD contains two EF-hands and a calmodulin-binding motif (CBM) [3, 21, 22]. The RD is connected with the ABD core via a linker [23]. PLS3 consists of 630 amino acids and the molecular mass of the protein is 70.8 kDa. In humans, the homology between PLS1 and PLS2 is 73%, 75% between PLS1 and PLS3, and 79% between PLS2 and PLS3 [3]. Comparisons of the plastin isoforms between humans and mice show 94% identity for PLS1, 96% for PLS2, and 99% for PLS3 [3].

Plastins cross-link actin filaments into bundles, which are higher-order assemblies, through the tandem pair in their ABDs [24, 25]. So far, only the crystal structures of the ABD1 and ABD2 actin-binding cores are resolved [24, 26]. A homology-based model of the PLS3 actin-binding core has been generated using the Phyre2 web portal for protein modeling, prediction, and analysis [23]. The full-length crystal structures of all plastins remain to be determined.

### F-actin dynamics/ cell motility

The main function of plastins is the binding and bundling of F-actin [3, 23, 27, 28]. F-actin is a polymer composed of globular (G-actin) subunits. These microfilaments are the main components of the cytoskeleton [29]. The actin cytoskeleton is a complex network whose dynamic formation influences numerous fundamental physiological cellular processes, such as focal adhesion, cell motility, endo- as well as exocytosis, mechanotransduction and cell division [30]. The F-actin network underlies a tight regulation and must be assembled, maintained, and disassembled in 3D at the correct time and place, and with proper filament organization and dynamics [31]. Thereby, the dynamic formation of the actin cytoskeleton is dependent on various F-actin-binding and -bundling proteins [32].

Plastins are involved in the assembly and organization of the actin cytoskeleton through their two ABDs. Binding of each ABD to two separate actin filaments promotes the formation of a bundle with a center-to-center distance of ~ 120 Å between the filaments [28]. However, the exact mechanism by which the ABD1 and ABD2 bind and bundle actin is poorly understood. It is assumed that both ABDs differently interact with actin [26]. Actin-binding is mediated through ABD1, while bundling is facilitated through both ABDs [33]. Furthermore, the plastin isoforms differentially interact with F-actin resulting in distinct F-actin organization [17, 20]. It has been suggested that PLS3 binding via both ABD1 and ABD2 is essential at the leading edge and focal adhesions [23]. The F-actin-binding and -bundling activity of the ABD domains is Ca^2+^ dependent, as the binding of Ca^2+^ by EF-hands inhibits the interaction with F-actin [33, 34].

The ABD cores form a compact and rather globular structure in an antiparallel arrangement induced by the contact between the CH1 and CH4 domains at the N- and C-terminal ends [3, 26]. This conformational structure seems to be highly dynamic and dependent on the presence of Ca^2+^ [35]. The conformational plasticity of CH2, located within the structurally polymorphic ABD1, influences the diverse functions of different actin assemblies [22]. This indicates that the structural plasticity is related to the function of the plastin isoform. Furthermore, the crystal structure indicates that the two ABDs harbor different binding characteristics with F-actin [20, 33].

As an F-actin-binding and -bundling protein, PLS3 plays an essential role in cell motility [36]. The movement of a cell is activated by adhesion to the extracellular matrix, executed through membrane remodeling at the leading edge and controlled by the actin cytoskeleton [37, 38]. PLS3 promotes membrane protrusion and cell migration to overcome gaps in the extracellular matrix and when adhesion is spatially gapped [39]. In the skin, PLS3 influences the basement membrane assembly [40]. Furthermore, PLS3 is involved in membrane trafficking under hypoxia [41]. All these functions arise from the interaction with the actin cytoskeleton. It has been speculated that PLS3 serves as a mechanical link between the actin polymerization network at the front of the cell and the myosin motor activity in the cell body [36]. PLS3 modulates the actin dynamics and generates force independent of cross-bridge formation mediated by actin related protein 2/3 complex (Arp2/3) [42]. The Arp2/3 complex facilitates the actin polymerization which is essential to generate pushing forces capable of deforming the cell membrane [31].

### Influence of intracellular calcium on PLS3-dependent processes

The crucial role of Ca^2+^ for F-actin-bundling by plastins has been a subject of research for a long time [43]. Especially, in terms of PLS2, studies focused on its Ca^2+^-dependent functions regarding actin-bundling and specifically T-cell activation and motility [34, 44], reviewed in Babich, Burkhardt [45], Morley [46].

Interestingly, comparisons of the three plastin isoforms show a much lower amino acid identity at the N-terminal EF-hand domains than the higher conserved actin-binding domains. Thus, several studies compared the plastin isoforms regarding their Ca^2+^ sensitivities and Ca^2+^-dependent functions [33, 47–49].

EF-hands of both PLS2 and PLS3 were shown to consist of alpha-helix-rich sequences and underlie conformational changes upon Ca^2+^-binding [47]. PLS3 has a lower sensitivity for Ca^2+^ as indicated by only a slight change of the EF-hands’ secondary structure in contrast to PLS2. Also, higher concentrations of Ca^2+^ are required for structural changes of the EF-hand motifs of PLS3 [47]. Congruently, monitoring the disassembly of plastin/F-actin bundles induced by Ca^2+^, PLS3 has been proven to be less sensitive to Ca^2+^ than both PLS1 and PLS2 [33]. Further analysis has shown a difference in affinity to Ca^2+^ within the two EF-hands of PLS3 (one high and one low affinity site) in contrast to PLS2 comprising two high-affinity sites [47].

Generally, the presence of Ca^2+^ reduces the ability to bundle F-actin in all three isoforms [33]. Further studies on the functional linkage between the EF-hands and the linker region CBM revealed inhibition of rapid proteolytic cleavage at the CBM upon Ca^2+^ stimulation, emphasizing a Ca^2+^-dependent linkage of these two regions in all three human isoforms. Focusing on the impact of Ca^2+^ on the ABDs, inequality is found: one ABD, most likely ABD1, binds F-actin independent of the presence of Ca^2+^; whereas at the other ABD, most likely ABD2, F-actin-binding is inhibited by Ca^2+^ bound by the EF-hands [33].

To gain further insight into the process of Ca^2+^-binding to the EF-hands as well as into kinetic and structural details on Ca^2+^-regulated domains of plastins, three biophysical methods have been used: surface plasmon resonance studies, isothermal titration calorimetry studies, and nuclear magnetic resonance spectroscopy [48]. In terms of Ca^2+^-regulated function, a crucial role of the regulatory helix 5 (H5) linker region localized between the second EF-hand motif and the first CH1 domain of ABD1 has been suggested, which may even differ between the plastin isoforms. Precisely, the H5 region of PLS3 (TPL-EF-H5 construct) is not displaced by the bee venom peptide melittin, unlike a possible displacement in PLS2 (LPL-EF-H5 construct) [48].

Another approach assessed the Ca^2+^-coordination structures of PLS2 and PLS3 and their synthetic peptide analogs by Fourier transform infrared spectroscopy [49]. Based on the results, an association between the aggregation tendency of the two Ca^2+^ binding sites of PLS3 and its lower sensitivity to Ca^2+^ was suggested [49].

The importance of well-regulated Ca^2+^ homeostasis for the proper function of PLS3 is underlined by several findings of disturbed Ca^2+^ regulation associated with loss of its functionality, shown in PLS3 rescue in SMA models [50] or osteogenesis imperfecta (OI)-associated PLS3 mutations [23].

### Endocytosis

The role of PLS3 in endocytosis has been first described in budding yeast [51]. Knockout of the *PLS3* ortholog, Sac6, causes massive impairment of endocytosis; especially of the receptor-mediated internalization of the pheromone α-factor [51]. Actin-bundling is a crucial process required for proper endocytosis in which Sac6-mediated bundling of actin filaments forms a framework in the early stages of this process [52]. Actin organization in cortical actin patches, which are dynamic actin structures within the inner faced layer of the cell membrane in yeast, dependent on actin-bundling proteins Sac6 and Scp1 (ortholog of human SM22). Loss of both proteins results in tremendous defects in patch biogenesis (increased patch lifetime), while Sac6 loss alone causes random movement or patch disassembly, with the absence of fast movements [52]. In filamentous fungi, *Ashbya gossypii* and *Aspergillus nidulans*, the polarized hyphal tip growth and endocytosis are Sac6/fimbrin A dependent, and their lack causes reduced rates of endocytic uptake [53, 54]. This highlights the importance of PLS3 orthologs as actin regulators and the requirement of functional actin dynamics to fulfill proper extension growth across species. Additionally, Sac6 along with other actin-binding and -bundling proteins (such as Abp85) are required for the uptake of the maltose transporter in yeast [55]. Moreover, fimbrin cross-linkers, twisting the actin filaments, provide approximately one-sixth of the energy required for endocytosis [56, 57]. Importantly, Arp2/3-actin networks are crucial to generate the force during membrane invaginations. Those networks are disrupted in Sac6-deficient cells and consequently result in disturbed endocytosis [56].

In fission yeast, it has been proposed that fimbrin selectively regulates the access to actin filaments for other actin-binding proteins, such as the tropomyosin Cdc8p [58]. Accordingly, in fimbrin-deficient cells, mislocalization of tropomyosin to actin patches results in increased patch lifespan and decreased motility [58]. Fimbrin is also able to displace tropomyosin from actin filaments lowering the inhibition of myosin I by tropomyosin and thereby ensuring motor activity [59].

The role of PLS3 in endocytosis caught first attention in human studies when PLS3 has been identified as a protective modifier of SMA [5]. In the context of SMA, both F-actin levels and Ca^2+^- homoeostasis are reduced, two processes crucial for endocytosis, but restored by *PLS3* overexpression [5, 60–64]. Importantly, endocytosis is a key process in neurons as they rely on constant refilling of the recycling vesicle pool in the presynapse. Apart from other reasons, endocytosis is a main cellular process disturbed in SMA, contributing to disturbed function of neuromuscular junctions (NMJs) [61, 65–68].

Another approach assessed defects in endocytic pathways in a SMA model and hypothesize that PLS3 increases the expression of endocytic proteins by supporting the availability of ribonuclear protein granules containing the required components for their translation [68]. Local axonal translation is dependent on transport of mRNAs and microRNAs along the axons to the growth cone of polarized neurons. Thereby, mRNAs and microRNAs not only hitchhike on late endosomes and lysosomes but they also act as hubs for local translation, finally contributing to the function and maintenance of neurons and neuronal circuits [69, 70]. Congruently, PLS3 directly interacts with activated RAB5, an early endosomal protein, and thereby regulates endocytic activity in mammalian cells [71]. It is, therefore, tempting to postulate that *PLS3* overexpression may restore impaired vesicle trafficking in SMA and consequently local translation.

### Signaling

The dynamic regulation of F-actin assembly and the interaction with the actin cytoskeleton is regulated by the coordinated activation of actin assembly factors through different signaling cascades [31]. It is accepted that PLS3 is involved in these signal transduction pathways [72]. Early studies show that the up- or down-regulation of PLS3 influences different signaling pathways. Downregulation of PLS3 has been shown to inhibit the p38 mitogen-activated protein kinase (MAPK) signaling pathway in MDA-MB-231 cells, which mediates apoptosis [73]. In keratinocytes, PLS3 is involved in the calcineurin/nuclear factor of activated T-cell (NFAT) pathway, which is a major regulator of cell migration [74]. In osteocytes, PLS3 levels regulate the expression of RELA proto-oncogene, NFΚκB subunit. PLS3 interacts with NFκB repressing factor (NKRF) facilitating its translocation into the nucleus, and thus the transcription of nuclear factor-activated T cells c1 (NFATC1) and an important factor in osteoclastogenesis [16]. Through the PI3K/AKT signaling pathway, PLS3 regulates tumor progression by promoting the proliferation and migration of cancer cells [75].

Several studies have demonstrated that PLS2 can be phosphorylated at positions Ser 5 and Ser7 during leukocyte activation by various stimuli [3]. Phosphorylation of Ser5 is mediated by cAMP-dependent protein kinase A, while the relevant kinase for Ser7 is still controversially discussed. In osteoclasts, phosphorylation of PLS2 on Ser5 and Ser7 increases the F-actin-bundling capacity [76] and the avidity for cellular F-actin and F-actin-binding activity [77]. In macrophages, PLS2 is phosphorylated exclusively on Ser5 by stimulation with bacterial lipopolysaccharide [78]. Ser5 is only found in PLS2 and not conserved in other plastin isoforms. To date, no phosphorylation of PLS3 is known and further studies are required [72].

## Disease involvement

PLS3 levels require a tight balancing in the cell. Aberrantly increased levels are associated with cancer and osteoarthritis. Instead in several neurodegenerative conditions associated with decreased F-actin levels, PLS3 overexpression acts protective. Instead, deletions or loss-of-function mutations in PLS3 cause osteoporosis in humans and mice. Finally, S-nitrosylation of PLS3 associates with thoracic aortic dissection (Fig. 1). Moreover, PLS3 is involved in infection and pathogen entry.

## Cancer

### Malignancies in solid tissues

A rising number of biomedical studies investigated PLS3 in the context of the most common malignancies (colorectal, prostate, breast, gastric, and lung cancer) and explored its potential use as a biomarker. Metastases in distant organs are the most common cause of death in these solid tissue tumors. The cell migration and invasion of tumor cells are driven by the modulation of the actin cytoskeleton. *PLS3* is highly expressed during epithelial–mesenchymal transition (EMT) in circulating tumor cells (CTCs) in colorectal cancer. Elevated levels of *PLS3* during EMT in CTCs negatively regulate expression levels of epithelial genes, and positively regulate the levels of mesenchymal genes and thereby increase the invasiveness of colorectal cancer [79]. Several mechanisms that could explain the regulation of *PLS3* in CTCs had been suggested. Chromosomal instability, a hallmark of cancer formation, can trigger a copy number gain of Xq23 leading to *PLS3* overexpression [79, 80]. A study in colorectal cancer cells states that *PLS3* is a downstream target of Lamin A, which is a risk factor for this type of cancer. *PLS3* in turn downregulates E-cadherin leading to increased invasiveness during EMT [81]. Furthermore, genetic polymorphisms modifying *PLS3* levels have been discussed. The intronic polymorphism rs871773 C > T is associated with a low recurrence time of colorectal cancer, while the rs6643869 polymorphism correlates with the time of tumor recurrence in women and has been identified in CTCs [82–84].

*PLS3* overexpression is found in two-third of CTCs from breast cancer patients in different stages of EMT and is associated with poor overall survival, especially in patients with luminal type A or triple-negative breast cancer [85]. Silencing of *PLS3* in MDA-MB-231 triple-negative breast cancer cells increases the sensitivity towards the anti-cancer agent paclitaxel [73]. A recent study has published a gene panel consisting of seven genes (*PLS3*, *MGB1*, *HER2*, *CK19*, *CDH1*, *CDH2*, and *VIM*) that can be used to discriminate EMT stages in CTCs tested by qPCR [86]. For prostate cancer, a gene panel with 14 genes has been developed including *PLS3*, *VIM*, and *CDH2,* and in 9.5% of patients an increased expression of *PLS3* has been found [87]. Interestingly, *LOXL1* downregulates *CDH1* and upregulates *VIM*, *CDH2*, *PLS3*, and *SNAI2* in gastric cancer cells, where expression of *LOXL1* correlates with EMT [88]. In gastric cancer, increased expression of *PLS3* indicates a poor prognosis and is associated with cancer differentiation, the depth of tumor invasion and EMT [89]. Chromatin immunoprecipitation (ChIP) assays in patients with gastric cancer have shown that the transcription factor *ZNF471* acts as a tumor suppressor gene. ZNF471 binds the *PLS3* promoter and suppresses its expression. Methylation of the CpG-methylation site of the *ZNF471* promoter is a useful prognostic marker for overall survival in gastric cancer patients [90]. High *PLS3* expression is also a feature of pancreatic cancer cells. Here, *PLS3* acts as an oncogene and its overexpression triggers the activation of the PI3K/AKT signaling pathway leading to cancer progression [75]. In non-small-cell lung cancer (NSCLC), increased PLS3 plasma levels are a predictor of poor survival. *PLS3* expression is of therapeutic value and can predict the responsiveness to treatment with Nivolumab—a PD-1 monoclonal antibody—in NSCLC patients [91]. Moreover, UV-light- and cisplatin-resistant tumors show elevated *PLS3* expression [92, 93]. Downregulation of *PLS3* in human liver cancer cells increased the sensitivity of these cells to cisplatin [94]. Cells lacking *PLS3* expression are sensitive to DNA damage and silencing of *PLS3* led to increased damage by UV light. By this, expression of *PLS3* could be used as a therapeutic marker in irradiation therapy [94]. Together, the mentioned studies suggest an association of increased *PLS3* expression during EMT and a high prognostic potential of *PLS3* as a biomarker in malignancies of solid tissues. The expression levels of *PLS3* represent a useful marker for cancer prognosis especially as part of a gene panel.

### Malignancies of the hematopoietic and lymphatic system

Elevated *PLS3* expression is a negative prognostic marker for acute myeloid leukemia (AML), the most common acute leukemia in adults, while knockdown of *PLS3* increases survival in vivo [95]. In Sézary Syndrome (SS), an aggressive, rare form of cutaneous T-cell lymphomas (CTCL), circulating CD4^+^ T-cells show increased expression of *PLS3*, *TWIST1* and *GATA6* compared to normal CD4^+^ T-cells [96, 97]. The promoter regions of all three genes have been found to be hypomethylated in SS CD4^+^ T cells indicating an epigenetic regulation of the expression levels. This is of large interest, since all three genes are located on different chromosomes [98–101]. Therefore, *PLS3* has been suggested as a biomarker for SS CD4^+^ T cells and it is associated with an unfavorable disease outcome [102–104].

## Bone disorders

### Osteoporosis

Dynamic regulation of the actin cytoskeleton by actin-binding and -bundling proteins like PLS3 [3, 20] is particularly crucial in the musculoskeletal system to instantly adapt to environmental changes through mechanotransduction [105, 106]. Mechanotransduction is the conversion of mechanical signals into cellular response, which is assumed to play an essential role in several pathologies of the musculoskeletal system, such as osteoporosis [107, 108] and osteoarthritis [109–111]. In chicken, fimbrin is detected in the dendrites of osteocytes [105, 112], which play a key role in mechanotransduction [113]. Therefore, PLS3 could influence the mechanical signal transformation [108].

In 2013, pathogenic variants in *PLS3* have been reported to be associated with osteoporosis including fractures in men and mild osteoporosis in women [108]. Moreover, *PLS3* showed a significant association with osteoporosis in women upon menopause, suggesting that mutations in *PLS3* not only associate with the monogenic but also the complex genetic trait of osteoporosis [108]. Osteoporosis is a multifactorial disease that is dependent on hormonal, environmental as well as genetic factors [114, 115]. Low bone mineral density (BMD) seems to be genetically determined in 50–85% of cases [114, 116–118]. This emphasizes the genetic component as a determining factor for BMD and its relating fracture risk. Primary (hereditary) osteoporosis usually becomes symptomatic in childhood and is, therefore, referred to as early-onset osteoporosis. The clinical features are characterized by low BMD (age normalized average score (*Z*-score < 2.0)), occurring vertebral compression fractures (VCFs), or low-trauma fracture history [119, 120]. The most common form of monogenic osteoporosis is OI [120], which additionally affects extraskeletal features like blue sclera, joint hypermobility, and deafness [15, 114, 121]*.* Around 85–90% [15, 114] of OI cases are linked to a dysregulation in type I collagen, although the list of OI-associated genes is increasing [15, 114, 122–125].

**Fig. 3:**
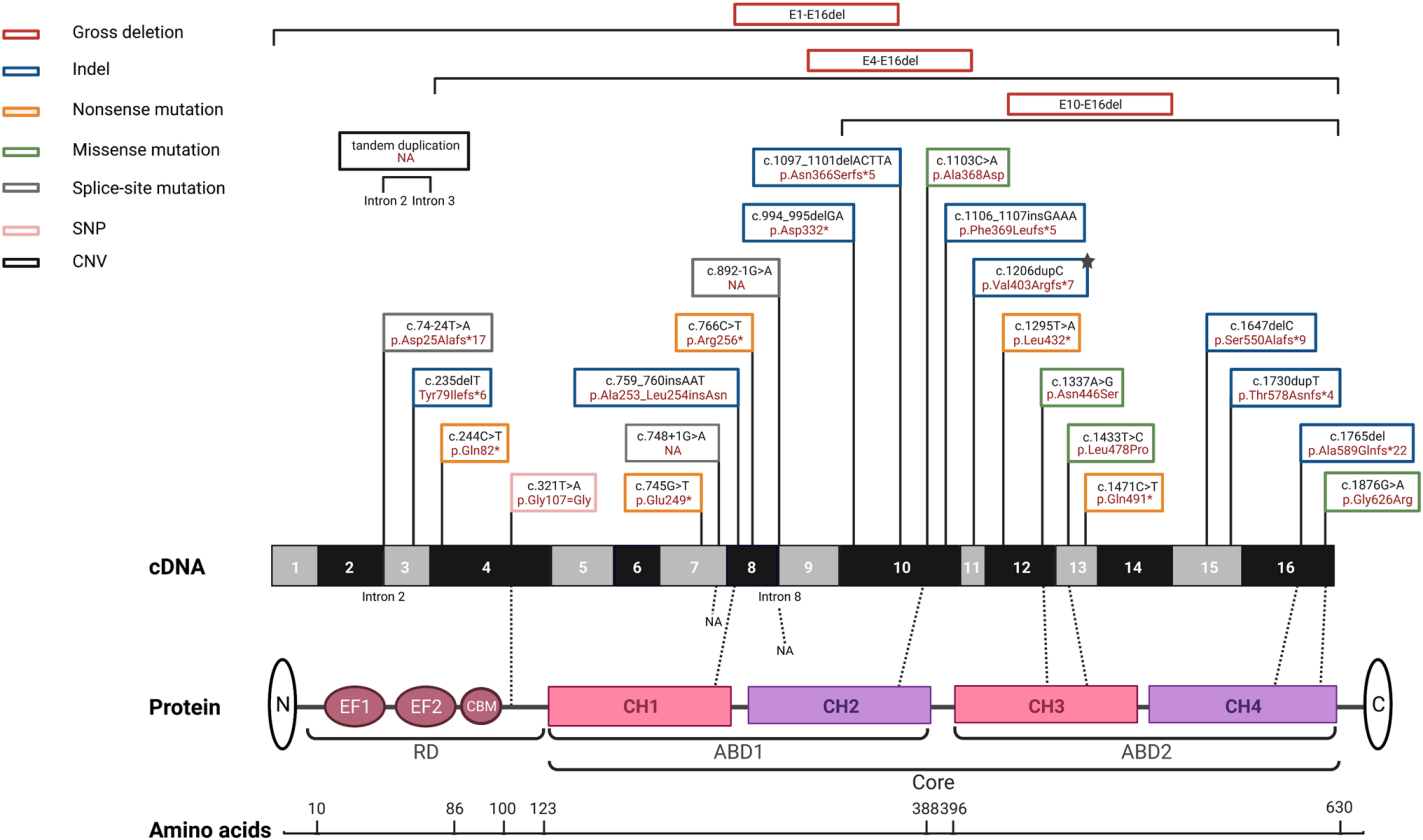
Schematic representation of the *PLS3* gene and corresponding protein domains. Illustrated are the causative mutations for osteoporosis and the resulting amino acid changes that were previously published. All depicted mutations were modified according to HGMD Professional (NM_005032.7 (GRCh38)). References and clinical characterization are shown in Table 2. Note, RNA containing frameshift and nonsense variants (except for the last exon 16), most likely undergo nonsense-mediated mRNA decay and thus no protein is produced. *E* EF-hand motifs, *CBM* calmodulin-binding motif, *RD* regulatory domain, *CH* calponin-homology domain, *ABD* actin-binding domain, *Core* actin-binding core domain, *SNP* single-nucleotide polymorphism, *CNV* copy number variation, *NA* not available; ★ = PLS3 isoform including 18 exons, accession No.: NG_012518. The figure was created with BioRender.com

Up to date, 27 mutations in *PLS3* are associated with early-onset osteoporosis (Table 2; Fig. 3). Nevertheless, rare variants also indicate OI traits [108, 126, 127], which is why *PLS3* was also included as a genetic cause for OI according to Van Dijk, Sillence [121]. However, several studies have reported variants that lead to severe skeletal abnormalities among women, resembling the osteoporotic phenotype of men [108, 115, 128–130]. This huge variation in heterozygous women is suggested to be caused by X-inactivation of the mutant allele or PLS3 escaping X-inactivation which is why women are less severely affected [15, 126, 129, 131, 132]. Even though bone morphometry was very heterogeneous [16, 126, 133–136], most male patients showed peripheral fractures, low BMD, VCFs, especially in the thoracic spine, and low bone turnover rate, while only a few developed extraskeletal OI traits [108, 126–128, 131, 137–140], developmental delay [15, 127] or neuromuscular abnormalities, like waddling gait [108, 126, 131, 141]. So far, no specific biomarkers have been identified, which can distinguish genetic factors of osteoporosis, although microRNAs are getting more and more popular as functional markers [120, 142]. This makes it difficult to diagnose *PLS3* mutations, although genetic analysis would be important to assess bone fragility risk within families. Thus, to find genetic causes of bone disorders, it is recommended to use gene panel screening containing known monogenic osteoporosis genes in fracture-prone children to identify genetic factors influencing bone health and to reveal family risk [114, 143–145].

**Table 2:** Clinical findings of patients with X-linked early-onset osteoporosis caused by *PLS3* mutations

ID	Reference	Patient (Term based on reference)	Exon/Intron	Variant	Amino acid change	Hemizygous						Heterozygous					
						Age of intervention	Age at first fracture (years)	Peripheral fractures minor traumas	VCFs	BMD (LS: *Z*-score)	Extraskeletal features (OI traits)	Age of intervention	Age at first fracture (years)	Peripheral fractures minor trauma	VCFs	BMD (LS: *Z*-score)	Extraskeletal features (OI traits)
1	[108]	1.III-1	E3	c.235delT	p.Tyr79Ilefs*6	–	–	–	–	–	–	45	Childhood	Yes (1)	None	− 0.1	Joint hyperlaxity
		1.III-2				32	2	Yes (10)	Yes	− 5.5	None	–	–	–	–	–	–
		1.III-4				–	–	–	–	–	–	40	–	None	NA	0.0	Joint hyperlaxity
		1.IV-1				21	8	Yes (1)	Yes	− 1.1	None	–	–	–	–	–	–
		1.IV-2				10	Childhood	Yes (6)	None	− 2.1	None	–	–	–	–	–	–
		1.IV-3				4	4	Yes (1)	None	− 3.2	Joint hypermobility	–	–	–	–	–	–
		1.IV-7				6	6	Yes (17)	None	− 3.7	Waddling gait, joint hypermobility	–	–	–	–	–	–
		1.IV-8				10	Childhood	Yes	None	− 2.4	Waddling gait, epilepsy, joint hypermobility	–	–	–	–	–	–
2		2.II-1	E13	c.1471C > T	p.Gln491*	–	–	–	–	–	–	59	52	Yes (3)	Yes	− 3.4 (T-score)	NA
		2.II-2				–	–	–	–	–	–	62	–	None	NA	− 1.8	NA
		2.II-3				–	–	–	–	–	–	48	6	Yes (4)	NA	− 1.5 (T-score)	NA
		2.II-4				–	–	–	–	–	–	55	NA	NA	NA	NA	NA
		2.III-3				36	Till 18	Yes (5)	None	− 2.8	None	–	–	–	–	–	–
		2.III-7				34	7	Yes (13)	Yes	− 3.4	None	–	–	–	–	–	–
3		3.II-1	E7	c.748 + 1G > A	NA	NA	NA	Yes	Yes	NA	None	–	–	–	–	–	–
4		4.II-1	E8	c.759_760insAAT	p.Ala253_Leu254insAsn	54	Adulthood	Yes (1)	Yes	− 2.5	None	–	–	–	–	–	–
5		5.II-3	E15	c.1647delC	p.Ser550Alafs*9	41	NA	Yes (10)	Yes	− 2.8	None	–	–	–	–	–	–
6		6.I.2	E4b	c.321 T > A	p.Gly107 = Gly	–	–	–	–	–	–	44	–	None	NA	− 0.6	None
		6.II-1				15	NA	None	NA	− 2.9	Joint hyperlaxity	-	–	–	–	–	–
		6.II-3				–	–	–	–	–	–	11	–	None	None	0.0	Joint hyperlaxity, hand and feet malformation
		7				20	4	Yes (9)	NA	Normal	None	–	–	–	–	–	–
		8				9	NA	None	NA	Normal	Joint hyperlaxity	–	–	–	–	–	–
		9				11	4	Yes (8)	Yes	− 4	None	–	–	–	–	–	–
		10				54	45	Yes	Yes	− 3.4	None	–	–	–	–	–	–
7	[131]	P1	E10	c.994_995delGA	p.Asp332*	7	2.5	Yes (4)	Yes	− 3.5	Clumsy gait, mild spastic cerebral palsy	–	–	–	–	–	–
		P2				3	2.2	Yes (1)	Yes	− 1.7	None	–	–	–	–	–	–
8		P3	E13	c.1433 T > C	p.Leu478Pro	6	5	Yes (2)	Yes	− 3.4	None	–	–	–	–	–	–

ID	Reference	Patient (Term based on reference)	Exon	Variant	Amino acid change	Hemizygous						Heterozygous					
						Age of intervention	Age at first fracture (years)	Peripheral fractures minor traumas	VCFs	BMD (LS: *Z*-score)	Extraskeletal features (OI traits)	Age of intervention	Age at first fracture (years)	Peripheral fractures minor trauma	VCFs	BMD (LS: *Z*-score)	Extraskeletal features (OI traits)
9	[129]	II-3 ^0^	I2	c.74-24 T > A ^1^	p.Asp25Alafs*17	62	7	Yes (3)	Yes	− 5.0 (T-score)	None	–	–	–	–	–	–
		II-4 ^0^				65	33	Yes (4)	Yes	− 4.0 (T-score)	None	–	–	–	–	–	–
		II-5 ^0^				–	–	–	–	–	–	59	5	Yes (8)	Yes	− 3.6 (T-score)	None
		III-2 ^0^				–	–	–	–	–	–	53	38	Yes (1)	None	− 1.9 (T-score)	None
		III-6				–	–	–	–	–	–	33	–	None	None	− 2.2	None
		III-7				–	–	–	–	–	–	40	–	None	None	− 2.7	None
		IV-1				35	21	Yes (1)	Yes	− 1.9	None	–	–	–	–	–	–
		IV-2				33	7	Yes (10)	Yes	− 2.7	None	–	–	–	–	–	–
		IV-6				12	8	Yes (4)	Yes	− 2.7	None	–	–	–	–	–	–
		IV-7				9	8	Yes (2)	Yes	− 3.1	None	–	–	–	–	–	–
10	[127]	Patient 2	E10	c.1103C > A	p.Ala368Asp	12	NA	Yes	NA	− 5.6 (whole body)	Deafness, blue sclerae, small joint laxity, facial dysmorphism (other gene mutations)	–	–	–	–	–	–
		Patient 3				7	NA	Yes (1)	NA	− 4.2 (whole body)	Deafness, blue sclerae, small joint laxity, facial dysmorphism (other gene mutations)	–	–	–	–	–	–
11	[138]	1.III-1	entire gene	3.411-MB deletion	NA	10	4	Yes (4)	Yes	− 2.1	NA						–
		1.III-2				–	–	–	–	–	–	9	–	None	None	− 1.1	NA
		1.III-3				–	–	–	–	–	–	8	–	None	None	− 0.5	NA
		1.II-5				–	–	–	–	–	–	NA	30	Yes (1)	None	NA	NA
12	[138]	2.III-1	E15	c.1730dupT ^2^	p.Thr578Asnfs*4 ^2^	4	2	Yes (2)	Yes	− 4.0	Grey sclerae	-	–	–	–	–	–
		2.III-2				–	–	–	–	–	–	NA	–	None	None	− 3.8	Grey sclerae
		2.II-2				–	–	–	–	–	–	NA	–	None	None	− 1.3	Grey sclerae
13	[126]	F1.1	E4-E16	E4-E16 del	NA	9	NA	Yes	Yes	− 3.4	Facial dysmorphism, waddling gait	–	–	–	–	–	–
		F1.2				6	NA	Yes	Yes	− 3.4	Facial dysmorphism, waddling gait, small joint laxity, opalescent teeth	–	–	–	–	–	–
14		F2.1	E1-E16	E1-E16 del	NA	12	4	Yes (7)	Yes	− 3.6	None	–	–	–	–	–	–
15	[128]	Cohort I—Patient 1	E8	c.766C > T	pArg256*	18	9	Yes (4)	Yes	− 4.1	Blue sclerae, joint hyperlaxity, teeth problems	–	–	–	–	–	–
		Cohort I—Mother				–	–	–	–	–	–	46	35	Yes (1)	NA	− 1.4	Blue sclerae, joint hyperlaxity
16		Cohort I—Patient 2	E12	c.1337A > G^3^	p.Asn446Ser	–	–	–	–	–	–	10	6	Yes (4)	Yes	− 6.6	Joint hyperlaxity
17	[146]	FM-1	E10-E16	E10-E16del	NA	10	2	None	Yes	− 3.0	None	–	–	–	–	–	–
		Mother				–	–	–	–	–	–	47	NA	NA	None	− 1.4 (T-score)	None
18	[144]	Patient 1	E16	c.1765delG	p.Ala589Glnfs*22^4^	38	12	Yes	Yes	− 4.8	None	–	–	–	–	–	–
19		Patient 2	E12	c.1295 T > A	p.Leu432*	15	2	Yes	Yes	− 2.7	NA	–	–	–	–	–	–
20	[139]	P4	I8	c.892-1G > A	NA	11	4	Yes (5)	None	− 1.8	Joint hyperlaxity	–	–	–	–	–	–
21	[140]	IV-1	E7	c.745G > T	p.Glu249*	11	6	Yes (2)	Yes	− 1.2	Blue sclerae	–	–	–	–	–	–
		III-4				–	–	–	–	–	–	40	NA	None	None	0.0	NA
22	[122]	9	E11^5^ (isoform)	c.1206dup	p.Val403Argfs*7	NA	13	Yes	Yes	− 2.3	NA	–	–	–	–	–	–
23		10	E16 ^6^	c.1876G > A	p.Gly626Arg	NA	18	Yes	Yes	− 3.9	NA	–	–	–	–	–	–
24	[137]	Patient	E10	c.1097_1101delACTTA^7^	p.Asn366Serfs*5^7^	6	2	Yes	Yes	− 3.5	Blue sclerae, joint hypermobility, hearing loss, dysmorphic features	–	–	–	–	–	–
25	[147]	Patient	I2-I3	tandem duplication in intron 2–3	NA	21	Childhood	Yes (10)	Yes	− 3.1	NA	–	–	–	–	–	–
		Brother				7	NA	Yes (3)	Yes	low	NA	–	–	–	–	–	–
		Mother				–	–	–	–	–	–	NA	NA	NA	Yes	Low	NA
26	[135]	Proband	E10	c.1106_1107insGAAA	p.Phe369Leufs*5	12	4	Yes (1)	Yes	− 2.0	Blue sclerae	–	–	–	–	–	–
		Brother				6	5	Yes (2)	None	− 0.2	Blue sclerae	–	–	–	–	–	–
		Mother				–	–	–	–	–	–	36	-	None	None	− 0.5	Blue sclerae
27	[130]	II-4	E4	c.244C > T	p.Gln82*	–	–	–	–	–	–	65	NA	Yes (2)	Yes	− 4.8	None
		III-6 (unaffected carrier)				–	–	–	–	–	–	41	NA	NA	None	0,4	None
		III-8				36	Till 10	Yes (1)	Yes	− 3.7	None	–	–	–	–	–	–
		IV-2 (unaffected carrier)				–	–	–	–	–	–	17	NA	NA	None	-1.5	None
		IV-5				14	7	Yes	None	− 2.6	None	–	–	–	–	–	–

All mutations were modified according to HGMD Professional (NM_005032.7 (GRCh38)). Deviations are provided in the footnotes

^0^Post bisphosphonate treatment

^1^In [129]: c.73-24 T > A

^2^In [138]: c.1730dup, exact frameshift not mentioned in paper

^3^In [128]: c.1424A > G

^4^In [144]: p.Ala589fs

^5^In [122]: PLS3 isoform with 18 exons

^6^In [122]: variant located on E18 of PLS3 isoform with 18 exons, NG_012518

^7^In [137]: c.1106_1107insGAAA, p.Phe369Leufs*5

In summary, most of the osteoporosis-related *PLS3* variants are frameshift [108, 122, 135, 137, 138, 144] or nonsense mutations resulting in premature termination codons [108, 128, 130, 140], which are followed by mRNA decay [108, 128, 131, 135]. Next to that, partial deletions within *PLS3* were identified [126, 140, 146]. Surprisingly, no phenotypic differences were seen between mutations causing deletion or truncation of PLS3 protein or missense or in frame insertion mutations resulting in mutated PLS3 protein, most likely because all types of mutations cause a loss of function of PLS3 [108, 128–131, 135, 140, 147].

Studies of missense mutations in full-length PLS3 report impairments in F-actin-bundling ability or defects in Ca^2+^ sensitivity, which lead to localization shifts within the cell and can also disturb Ca^2+^-dependent structural rearrangements necessary for actin dynamics [23]. Impaired actin dynamics caused by mutations within the ABD2 of PLS3 result in inefficient F-actin-bundling properties, without influencing actin-binding by ABD1 [23]. In turn, this specific mutation interferes with PLS3′s actin-cytoskeletal association and thus, its localization at the lamellipodia and leading edge [23]. Considering these findings, it could be hypothesized that the proper intracellular localization of PLS3 is dependent on its actin-bundling function [23]. Similarly, in another study, a *PLS3* mutation, disrupting the ABD interface, results in conformational changes and defective actin-binding and -bundling function [140].

A cellular mislocalization and impaired Ca^2+^-dependent actin-associated distribution of PLS3 were also related to mutations influencing the Ca^2+^ sensitivity, causing either hypo- or hypersensitivity to Ca^2+^. Misregulated PLS3 cycling could thereby result in impaired bone homeostasis and might explain the osteoporotic phenotype in these patients [23].

To analyze the proposed functions of PLS3 regarding mechanotransduction [5, 15, 108, 120, 143, 144, 148], Ca^2+^ regulation [23, 33, 149]*,* osteoblastic bone mineralization [15, 120, 126, 131, 134, 143, 144, 150], osteoclastogenesis [16] and vesicular trafficking [15, 16, 61, 151], various *PLS3* mutations have been investigated and mouse model studies established to specifically investigate the effect of PLS3 loss or overexpression [16].

It is well established that PLS3 is highly abundant in the dendrites of osteocytes, which not only control mechanosensing but also regulate osteoblast and osteoclast activity, and an impairment of this cell system might explain the resulting osteoporotic phenotype [136, 152]*.* Indeed, in osteocytes, mutations in PLS3 interfere with cellular signaling, resulting in imbalanced bone homeostasis [121, 129]. In line with this, data acquired from PLS3 mutation carriers showed increased levels of apoptotic osteocytes together with abnormal gene expression of osteocyte-related genes [153]*.*

Because *PLS3* mutations cause childhood-onset osteoporosis associated with low BMD, it has been further hypothesized that the bone-forming osteoblasts might be disturbed by PLS3 loss [129, 143]. In healthy bone conditions, PLS3 abundance increases upon osteoblast differentiation [131, 154]*.* However, in some patients with *PLS3* mutations, matrix mineralization and osteoblast number are decreased [126, 129, 131, 155]. The resulting imbalanced levels of osteoblasts and osteoclasts, favoring bone resorption, might result in osteoporosis.

For differentiation and bone formation, osteoblasts rely on high Ca^2+^ concentrations, which is in part regulated by the interaction of the Ca^2+^-binding proteins PLS3 and PLS2. Through their interaction, bound Ca^2+^ is released to increase the intracellular Ca^2+^ level when the extracellular Ca^2+^ level is low [149]. However, mutations in Ca^2+^ binding domains (EF-hands) of PLS3 weakened the interaction of PLS3 with PLS2, as revealed by PLS2-TRAP co-immunoprecipitation and western blot analysis, and thus might contribute to the osteoporotic phenotype [149].

To enable further analyses, a ubiquitous *Pls3* KO mouse model has been generated that is comparable to loss-of-function mutations observed in OI patients [16]. In 3-month-old *Pls3* KO animals reduced cortical and trabecular thickness and decreased bone parameters is seen. In line with humans, the osteoporotic phenotype in male mice is more pronounced than in females [16]. Importantly, the NFκB pathway, an essential pathway for osteoclastogenesis, is strongly influenced upon PLS3 loss [16]. NFκB signaling is initiated through binding of receptor activator of NFκB ligand (RANK-L) to its receptor RANK on the surface of osteoclasts, activating the transcription of nuclear factor-activated T cells c1 (*Nfatc1*) [156]. Besides, *Nfatc1* expression is negatively regulated by the NFκB-repressing factor, NKRF. As PLS3 binds to NKRF, *Pls3* KO results in insufficient repression of *Nfatc1* nuclear translocation, and therefore in increased osteoclast differentiation [16]. Moreover, podosomes, specific F-actin ring structures in osteoclasts, important for resorptive activity, migration, and adhesion, are structurally disturbed in *Pls3* KO mice, which might be due to increased depolymerization of F-actin and, thus, instability of the cytoskeleton [16].

When investigating the effects of elevated PLS3 levels in mice carrying homozygously a human *PLS3* transgene under an ubiquitously expressing promotor and integrated into the *Rosa26* locus on chromosome 6, a thickening of the cortical bone is seen in 3-month-old female *PLS3* OE mice compared to the controls [16]. This led to increased stiffness and breaking force in the females, which might be due to impaired bone renewal [16, 116]. In contrast to *Pls3* KO, *PLS3* OE might cause increased translocation of NKRF to the nucleus, which in turn results in inhibition of *Nfatc1* transcription and consequently inhibits osteoclastogenesis [16]. These PLS3 effects might be additionally influenced by impaired intracellular vesicle dynamics [61]. Similar to the *Pls3* KO, also in *PLS3* OE mice defective podosome formation is detected, probably caused by reduced F-actin disassembly [16]. In summary, it is assumed that an optimal amount of PLS3 is essential to sustain bone health. Neither removal of PLS3, resulting in bone abnormalities, nor an increased level of PLS3, leading to a hyperosteotic phenotype, seems to be beneficial for bone homeostasis but rather detrimental.

### Osteoarthritis

In 2015, increased PLS3 levels have been reported in chondrocytes from patients with osteoarthritis compared to healthy individuals [157]. Osteoarthritis is a multifactorial disease that affects the whole joint and is defined by degeneration of the articular cartilage, synovial inflammation and increased bone mass in the subchondral bone [158]. This is in line with the observed elevated cortical thickness in *PLS3* OE mice, which might be due to inhibited osteoclastogenesis [16]. Furthermore, the RANKL–RANK pathway plays a crucial role not only in osteoarthritis in general [159–161], but seems to be stressed in osteoarthritis patients showing increased PLS3 levels [157]. Further, differentially upregulated pathways in osteoarthritis patients are related to the actin cytoskeleton, endocytosis, TGF-β, MAPK, TNF-α, WNT and general metabolism processes [157]. As mentioned before, an interference of the mechanotransduction process is a crucial factor associated with the pathogenesis of osteoarthritis, where also the impact of PLS3 on the actin cytoskeleton is suggested to play an essential role [109–111]. Recent findings have shown that patients with varus gonarthrosis-induced osteoarthritis show higher levels of PLS3 in the medial compartment compared to the less affected lateral knee compartment. Besides, PLS3 was also predominantly found at the direct force-facing contact side of the respective joints [162]. These data might confirm previous assumptions that PLS3 seems to be involved in the mechanotransduction system and its enhanced level is associated with osteoarthritis. So far, less is known about the effect of *PLS3* deletions on cartilage. Another study has shown no effect on the intervertebral disc of osteoporosis patients linked to loss of function and *PLS3* deletions [148]. The influence of *PLS3* overexpression, *PLS3* loss, and *PLS3* mutations on articular cartilage itself remains elusive and the underlying mechanism affecting cartilage health still needs to be explored.

Since about 5% of the general population show increased *PLS3* expression in blood [5], it would be interesting to study if the increased expression of *PLS3* in blood is associated with an increase of PLS3 in cartilage, that seems to be associated with osteoarthritis, and thus can act as a biomarker for osteoarthritis as well [162].

## Thoracic aortic dissection

In terms of its role in diseases, PLS3 has also been shown to be involved in the causative mechanisms of thoracic aortic dissection in mice [163]. In more detail, S-nitrosylation of PLS3, induced by angiotensin II and mediated by inducible nitric oxide synthase, promoted the formation of a complex of PLS3 with cofilin, which depolymerizes actin and facilitates actin dynamics, and plectin, which links various cytoskeletal proteins and is important in formation of cell junctions in endothelial cells. This process was detected to aggravate the development of thoracic aortic dissection via pathological angiogenesis as well as disruption of the adherent junctions at the endothelial barrier [163].

## Neurodegeneration

PLS3 is highly abundant in certain areas of the brain especially in the hippocampus but also in the spinal cord [164]. It is strongly upregulated during the development and maturation of motor neurons pointing towards an important role in these processes [66]. PLS3 is an important interaction partner of several proteins or protein complexes involved in neurodegeneration disorders such as SMA [5, 61], ataxia [66], amyotrophic lateral sclerosis (ALS) [68], and Charcot–Marie–Tooth (CMT) [165] (Fig. 1).

### Spinal muscular atrophy

One of the most unexpected findings was the discovery that PLS3 overexpression acts as a protective modifier of SMA [5]. SMA is primarily a neurodegenerative motor neuron disorder and the most common cause of genetic death in infancy. It is caused by deletions and functional loss of *SMN1*, while the copy gene *SMN2* strongly modulates the disease severity [166].

Rarely, individuals carrying a homozygous deletion of *SMN1* and three or four *SMN2* copies are fully asymptomatic, in contrast to approximately 99% of individuals with such a genotype who develop SMA. This suggests that protective modifier(s) in the genome of these subjects counteract the detrimental effect of reduced SMN levels [5, 65, 167]. PLS3 is the first human SMA protective modifier identified by transcriptome analysis of differentially expressed genes. All asymptomatic, but none of the symptomatic siblings of six discordant families showed an up to ~ 40-fold upregulation of *PLS3* in lymphoblastoid cell lines (where *PLS3* usually is not expressed). Instead, the same individuals show no difference in PLS3 expression in fibroblasts, suggesting a tissue-specific regulation [5]. Generation of iPSCs from fibroblasts of two discordant families showed that the expression of PLS3 is highly elevated in asymptomatic individuals but not in symptomatic siblings [167]. This is also found in motor neurons (MNs) differentiated from iPSCs [167]. The mechanism behind is still not fully understood.

Moreover, three additional SMA protective modifiers, coronin-1C (CORO1C), a Ca^2+^-dependent protein involved in actin dynamics, and two Ca^2+^ sensor proteins, neurocalcin delta (NCALD) and calcineurin-like EF-hand protein 1 (CHP1) have been identified [61, 65, 66]. Similar to PLS3, CORO1C acts protective when upregulated, whereas NCALD and CHP1 act protective when being downregulated as shown in various animal models [61, 65, 66]. Importantly CORO1C and CHP1 interact with PLS3. All modifiers are involved in endocytosis and restore reduced endocytosis in SMA (see section of 2.4).

*PLS3* overexpression acts protective not only in humans with *SMN1* mutations but also in zebrafish, worm, fly and mouse SMA models [5, 61, 64, 168–170]. In all systems, PLS3 overexpression ameliorates or counteracts the major hallmarks of SMA pathology. Reduced SMN level impairs F-actin dynamics, which might be due to a disturbed transport of β-actin mRNA along the axons [63]. F-actin dynamics is pivotal for cellular integrity and is involved in cellular shape, migration, vesicular trafficking, RNA translation, and endocytosis, among others [171]. In highly polarized MNs, all these processes are particularly crucial. In severely affected SMA models, numerous F-actin-dependent processes including axonal growth, axonal connectivity at the NMJ, neurotransmission, F-actin caging, synaptic vesicle recycling as well as proprioceptive input at MN somata are reduced [60, 64, 172–174]. All these processes were either restored or ameliorated by *PLS3* overexpression as shown across various SMA animal models [61, 64].

Unexpectedly, while impaired NMJ function and motoric abilities are ameliorated in severely affected mice, survival is prolonged by only a few days [64]. To generate a more comparable situation as found in discordant SMA families (where 3–4 *SMN2* copies are present, but never only 2 copies as in the severe SMA mouse model), an intermediate-like SMA mouse phenotype has been generated by subcutaneous injection of a low dose of *SMN*-ASOs (Nusinersen, 30 µg at P2 and P3) [61]. This slightly increased SMN level rescues inner organ function and doubles the survival rate. These mice still die due to MN loss with 1 month of age. In contrast, additional overexpression of human *PLS3,* from a homozygous *PLS3* transgene [64], rescues MN and NMJ function and significantly prolongs survival (60% survive > 250 days; 30% > 400 days), clearly proving the protective effect of *PLS3* overexpression in an intermediate SMA mouse model [61]. Besides, a gene therapy approach using adenovirus-associated virus (AAV9)-PLS3, in combination with low dose SMN-ASO, ameliorates the SMA phenotype in mice and prolongs survival [175, 176]. These experiments provide strong evidence that PLS3 is a genuine protective modifier and highlight the power of combinatorial therapies in SMA.

Exo- and endocytosis are crucial for neurotransmission in neurons. To properly maintain neurotransmission, endocytic uptake is essential to replenish the recycling pool, which supplies vesicles to the readily releasable pool (RRP) [177]. At NMJs of severely affected SMA mice, the organization and number of docked vesicles on the presynaptic site are significantly reduced causing a decrease of neurotransmitter release. Moreover, the RRP size is significantly reduced in SMA, and the depletion and refilling time constants of this pool tend to be slower [64, 172, 178]. F-actin is essential in all types of endocytosis [179] and its inhibition reduces endocytosis in neurons under high frequency stimulation [180]. Indeed, reduced SMN levels dramatically decreased endocytosis in vitro, as well as the FM1-43 uptake at NMJ level in SMA mice. Instead, *PLS3* overexpression fully rescued endocytosis to similar levels as observed in controls [61]. Moreover, endocytic uptake of FM1-43 in the presynaptic terminal of NMJs upon electrical stimulation was significantly reduced in SMA and fully restored to control levels in SMA-*PLS3* OE mice [61].

### *Chp1*-associated ataxia

PLS3 is a genuine interaction partner of CHP1, a Ca^2+^ sensor protein [66]. CHP1 is ubiquitously expressed, but particularly abundant in neuronal tissues. CHP1 is a negative regulator of calcineurin, the most important phosphatase dephosphorylating the dephosphins involved in endocytosis [181]. Biallelic mutations in *CHP1* cause autosomal recessive spastic ataxia (SPAX9; MIM 618438) in humans and mice [182–184]**.** Moreover, *PLS3* overexpression in homozygous *Chp1* mutant mice delays but does not ameliorate the ataxic phenotype at an early disease stage by preventing axon degeneration of Purkinje neurons [185]. *PLS3* overexpression increases membrane targeting of NHE1 [185], an important binding partner of CHP1 [186] that is also associated with ataxia when mutated, at an early disease stage [187]. Thus, *PLS3* overexpression has a moderate protective effect on ataxia caused by *Chp1* depletion and demonstrates its potential as a cross-disease modifier [185].

### *SOD1*-associated amyotrophic lateral sclerosis

*PLS3* overexpression in a *C. elegans* model of ALS, carrying the dominantly inherited G85R SOD1 mutant, proved to be beneficial [68]. PLS3 increased locomotion rate, ameliorated pharyngeal pumping defects and counteracted sensitivity to paralysis by aldicarb. Contrary, on a wild-type *C. elegans* background, *PLS3* overexpression had a detrimental effect on locomotion rate, strengthening the observation that the balance of F-actin is essential and its overload in a cell or organisms may lead to the opposite effect [68]. Since ALS and SMA share a lot of common pathological features, as well as molecular and cellular commonalities, further investigation of a beneficial impact of PLS3 on other ALS-involved genes, *e.g., C9ORF72* would be very interesting.

## Infection and pathogen entry

Plastins are conserved from yeast to mammals. Within all species, they fulfill functions in actin dynamics rendering rapid actin rearrangements upon pathogen challenges in the host cell while harboring distinct functions within the pathogen itself. Pathogens such as *Salmonella typhimurium, Shigella flexneri* or *Trichomonas vaginalis* enter the host cells by remodeling the actin cytoskeleton [188–191]. *S. tysphimurium* can even enter nonphagocytic cells like endothelial cells, upon the stimulation of CDC42 and RAC-1, which activate signaling of the pathogen protein SipA and thereby increase PLS3′s bundling activity [188]. At the contact point of host cell and pathogen, this process triggers actin rearrangements while leading to the formation of nucleation zones. PLS3 is recruited to these zones and stabilizes F-actin filaments. Arising from those zones, elongated actin filaments form protrusions surrounding the pathogen [188, 189, 191]. Nucleated actin filaments are the source of G-actin through active depolymerization to fuel the leading tips for the construction of the protrusion [191]. Finally, the pathogen is indulged in a vacuolic structure and released into the cytoplasm of the host cell. During infection with the Hepatitis C virus (HCV), PLS3 has been shown to regulate pathogen replication. Knockdown of *PLS3* results in decreased viral replication and is, therefore, a potential target for HCV therapy [192].

Moreover, fimbrin facilitates active actin-cytoskeleton organizational changes to, e.g., enable amoebic movements and protrusion formation [190]. In *Rickettsia*, PLS3 together with profilin, capping protein, and cofilin determine the actin tail length required for motility during infection. In this species, actin bundles underlying tail formation resemble the organization of cell protrusions [193].

In the fungal pathogen, *Candida albicans*, the PLS3 ortholog Sac6 associates with oxidative stress responses. Sac6 negatively regulates cytosol–nucleus transport of the key transcription factor Cap1 and thereby the expression of oxidative stress response genes [194].

## Biomarker

Differential expression of *PLS3* is a frequent characteristic of CTCs as well as primary tumors, while usually being absent from the hematopoietic system. This renders the protein as well as the mRNA as immaculate biomarker candidates. In a recent study, a peptide library has been screened for affinity to *PLS3*-overexpressing cancer cells. They identify the peptide TP1 (KVKSDRVC) and develop a fluorescein isothiocyanate-labeled TP1 molecule that is able to identify *PLS3*-overexpressing CTCs in peripheral blood [195].

With regard to SMA, PLS3 is probably of limited use as a biomarker since it is only expressed in a minority of the human population in the hematopoietic system [5]. A study tested the expression of six putative protein biomarkers (COMP, DPP4, CLEC3B, SPP1, VTN, and AHSG) in mice with or without overexpression of a human *PLS3* transgene. The expression level of PLS3 did neither affect the amount of SMN nor did the other putative biomarkers, supporting the hypothesis that *PLS3* acts as an independent protective modifier in SMA [196].

One pitfall of PLS3 is that its differential expression is linked to multiple different diseases. Comorbidity of other PLS3-associated medical conditions should always be considered when an analysis of PLS3 as a biomarker in peripheral blood is performed. In addition to that, as an X-linked gene, a sex-specific expression bias towards women has been observed [5, 197].

## Conclusion

Despite PLS3 not being essential for the survival of a cell or organism, both conditions overexpression or knockdown of *PLS3* can influence many cellular processes either causing, facilitating or rescuing certain pathological phenotypes. While the full knockout in humans and mice causes a disruption of the bone remodeling cells and, thus, osteoporosis, its overexpression in some neurodegenerative disorders such as SMA or ALS, where F-actin levels are decreased, acts as a protective modifier. However, in wild-type condition, overexpression of *PLS3* is detrimental and causes osteoarthritis or facilitates cancer. *PLS3* overexpression is strongly correlated with several cancer types, and has been recommended for use as biomarker in cancer and poor prognosis for survival. PLS3 has an important function in F-actin-binding and -bundling and as such is involved in a plethora of cellular processes dependent on F-actin dynamics. These include cell migration and growth, axonal and neurite outgrowth of polarized cells, axonal local translation, endocytosis, influence of intracellular calcium on PLS3-dependent processes, mechanotransduction, signaling, infection with pathogens and others. This ever-growing knowledge on PLS3 is crucial to wisely use *PLS3* overexpression or PLS3 reduction as a therapeutic target.

## Abbreviations

ABD1: Actin-binding domain 1
ABD2: Actin-binding domain 2
ALS: Amyotrophic lateral sclerosis
AML: Acute myeloid leukemia
Arp2/3: Actin related protein 2/3 complex
BMD: Bone mineral density
CBM: Calmodulin-binding motif
CH: Calponin-homology
CHP1: Calcineurin EF-hand protein 1
CMT: Charcot–Marie–Tooth
CORO1C: Coronin-1C
CTC: Circulating tumor cell
CTCL: Cutaneous T-cell lymphoma
EMT: Epithelial–mesenchymal transition
F-actin: Filamentous actin
G-actin: Globular actin
HCV: Hepatitis C virus
iPSC: Induced pluripotent stem cell
MAPK: P38 mitogen-activated protein kinase
MN: Motor neuron
NCALD: Neurocalcin delta
NFATC1: Nuclear factor-activated T cells c1
NFκB: Nuclear factor 'kappa-light-chain-enhancer' of activated B cells
NKRF: NFκB repressing factor
NMJ: Neuromuscular junction
NSCLC: Non-small-cell lung cancer
OI: Osteogenesis imperfecta
PLS1: Plastin 1
PLS2: Plastin 2
PLS3: Plastin 3
RELA: RELA proto-oncogene, NFκB subunit
RD: Regulatory domain
SMA: Spinal muscular atrophy
SMN1: Survival of motor neuron protein 1
SMN2: Survival of motor neuron protein 2
SS: Sézary syndrome
VCF: Vertebral compression fracture
XCI: X-chromosomal inactivation
